# Short-chain fatty acid, butyrate prevents morphine-and paclitaxel-induced nociceptive hypersensitivity

**DOI:** 10.1038/s41598-023-44857-2

**Published:** 2023-10-18

**Authors:** Dawn Jessup, Kareem Woods, Sach Thakker, M. Imad Damaj, Hamid I. Akbarali

**Affiliations:** Department of Pharmacology and Toxicology, Virginia Commonwealth University, Richmond, 23298 USA

**Keywords:** Physiology, Neurophysiology, Addiction, Neuropathic pain, Peripheral neuropathies

## Abstract

Nociceptive hypersensitivity is a significant side effect with the chronic administration of opioids as well as chemotherapeutics. Both opioid-induced hypersensitivity (OIH) and chemotherapy-induced hypersensitivity (CIH) are characterized by an increased sensitivity to painful stimuli which can significantly reduce the quality of life for individuals on either drug(s). Here we demonstrate the nociceptive hypersensitivity associated with repeated administration of morphine (opioid) and paclitaxel (chemotherapeutic) treatment can be reversed by oral supplementation with the short-chain fatty acid (SCFA) sodium butyrate (NaBut). In two separate mouse behavioral models for nociceptive hypersensitivity, we found that thermal hyperalgesia (for OIH) and cold allodynia (for CIH) were prevented by treatment with oral butyrate (p.o, b.i.d). Electrophysiological recordings of small diameter dorsal root ganglia (DRG) neurons from morphine and paclitaxel treated mice showed an increase in neuronal hyperexcitability in both drug models which was likewise prevented by oral butyrate treatment. Using colonic conditioned media obtained from excised colon segments we found that gut mediators of morphine treated mice can induce hyperexcitability in naïve DRG neurons, but such enhanced excitability is not present when animals are co-treated with NaBut suggesting gut derived mediators modulate neuronal hyperexcitability. In-vitro NaBut treatment did not prevent morphine-induced excitability, suggesting an indirect role of butyrate in modulating neuronal hypersensitivity. These data taken together suggest that gut derived mediators affect opioid and chemotherapeutic-induced neuronal hypersensitivity that is prevented by the SCFA butyrate.

## Introduction

Chronic pain continues to be a major unmet clinical challenge affecting up to 30% of patients throughout their life^1,2^. Chronic pain can arise from many sources and can range from post-surgical pain, pain brought on by underlying pathologies, or even pharmacological interventions^1^. Opioid-induced hypersensitivity (OIH) and paclitaxel-induced peripheral neuropathy (CIPN) are common side effects of chronic opioid use and paclitaxel chemotherapy, respectively^3,4^. These conditions are both characterized by an increased sensitivity to painful stimuli and other sensory changes, which can significantly reduce the quality of life for individuals on opioids or chemotherapy. Despite the prevalence of OIH and CIPN, the underlying mechanisms of these phenomena are not fully understood^5,6^. However, recent studies have suggested that the gut microbiome may play a role in the development of both OIH and CIPN^7,8^.

The microbiome is the broad collection of microorganisms (bacteria, viruses, fungi, etc.) that reside in the gut, skin, and other tissues, and changes in the gut microbiome have been associated with a number of conditions such as fibromyalgia, rheumatoid arthritis, and irritable bowel syndrome^9^. Previous studies have shown that the gut microbiome can play a role in regulating pain by secreting or promoting the release of neurotransmitters, inflammatory cytokines, and other important messenger or regulatory signaling molecules^10^. We have previously demonstrated that the gastrointestinal microbiome is an important modulator of physiological and pharmacological effects induced by repeated administration of morphine (mor)^11^. Mice treated with an antibiotic cocktail showed significantly reduced gut bacteria and did not develop opioid induced increases in gut permeability, colonic mucosal destruction, inflammatory cytokine release or antinociceptive tolerance. Similarly, chronic morphine (25 mg pellet for 6 days) increased Gram-positive pathogenic bacteria and reduced bile-deconjugating bacterial strains in fecal samples resulting in an impairment of the gut barrier function and enhanced systemic inflammation^12^. These findings were recapitulated in primary afferent neurons isolated from dorsal root ganglia (DRG) innervating the lower gastrointestinal tract, wherein in-vivo administration of antibiotics prevented tolerance to morphine-induced neuronal hyperexcitability^11,13^.

The link between chemotherapy and the microbiome is likewise becoming increasingly apparent. In a study by Shen et al., the authors found that oxaliplatin-induced mechanical hyperalgesia was reduced in germ-free mice and in mice pretreated with antibiotics, and that restoring the microbiota of germ-free mice resulted in restoring the hyperalgesia from oxaliplatin. The authors concluded that these effects were likely being mediated in part by TLR4 expressed on immune cells including macrophages^14^. Similarly, a study by Ramakrishna et al.^8^ found that reciprocal gut microbiota transfers between a paclitaxel sensitive mouse strain (C57BL6/J) and a paclitaxel insensitive strain (129S6/SvEvTac) could confer the corresponding phenotype to paclitaxel-induced mechanical and cold hypersensitivity. The authors also found that paclitaxel decreased the abundance of certain bacterial species such as *Akkermansia muciniphila*, which was suggested to compromise gastrointestinal barrier integrity resulting in systemic exposure to bacterial metabolites and products that could then act upon the gut-immune-brain axis and alter neuronal function^8^.

Beyond changes in the microbial composition, many studies have also been done to investigate the varied effects that microbial metabolites have on host physiology^15–17^. We previously reported that media conditioned with colon segments isolated from chronic morphine treated mice induced tolerance and hyperexcitability in naive DRG neurons, and this effect was inhibited by oral vancomycin treatment^18^ demonstrating a link between pathogenic changes in the colonic microenvironment and neuronal outputs within the spinal cord. Perhaps the most well-established metabolites with the potential for therapeutic benefits in this regard have been the short-chain fatty acids (SCFAs) produced as a product of fermentation from dietary fibers by several species of gastrointestinal bacteria^19–21^. Propionate, acetate, and butyrate serve an essential role as an energy source for epithelial cells (enterocytes), modulate electrolyte and water absorption, and have been implicated as mediators of intestinal immune function. they may also have a role in maintaining neuronal and immune function along the gut-brain-axis, including along vagal nerve pathways^22,23^. Endogenous concentrations of SCFAs can vary greatly between tissues, and peripheral blood. For example, a previous study found concentrations in proximal colon can be around 70–140 mM while concentrations in peripheral blood were significantly lower at approximately 0.079 mM in human tissue^24^. Furthermore, loss of these fiber fermenting bacteria (by dysbiosis) has been suggested to play a role in the dysregulation of immune modulation, barrier integrity, and other homeostatic processes^20,22,25^. Indeed, butyrate in particular has emerged as a promising potential supplementary therapeutic in a number of inflammatory disease states^26–30^ including CIPN and opioid related side effects^31^.

In a recent study Cristiano and colleagues reported that sodium butyrate (NaBut) restored paclitaxel-induced altered gut barrier integrity, microbiota composition and food intake in a rodent model. Additionally, they reported that treatment with NaBut also ameliorated depressive- and anxiety-like behaviors induced by paclitaxel, and concluded the effects were associated with neuroprotective and anti-inflammatory mechanism^32^. Similarly, Cruz-LeBron and colleagues found that patients on methadone maintenance treatment (MMT) had significantly reduced levels of fecal SCFAs, and reduced abundance of *Akkermansia muciniphila* resulting in increased intestinal permeability consistent with their opioid induced dysbiosis^33^.

Collectively these studies have led us to conclude that a plausible link between the behavioral and physiological consequences of both opioid use and chemotherapeutic treatment may be similarly alleviated by way of addressing the underlying gastrointestinal dysbiosis. However less is known about the underlying neuro-epithelial changes in the presence of either drug and SCFA supplementation. Therefore, the goal of our study was to investigate if oral butyrate supplementation could efficaciously reduce both whole animal behavioral hypersensitivity as well as neuronal hypersensitivity at the level of the dorsal root ganglia and establish if these effects were the result of localized effects to the gastrointestinal tract.

## Results

### Repeated administration of morphine, or paclitaxel produce nociceptive hypersensitivity which can be prevented by treatment with sodium butyrate

**Figure 1:**
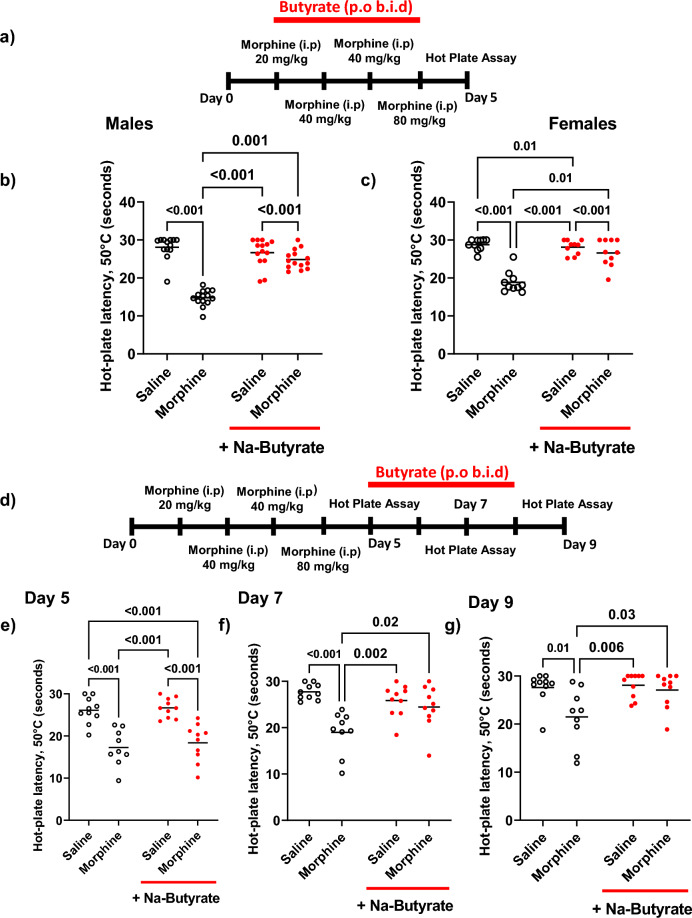
Repeated administration of morphine induces thermal hyperalgesia and is alleviated by NaBut. (**a**) Experimental timeline for development of opioid-induced hypersensitivity, (**b**, **c**). Mice were treated with a ramping dose of repeated administration of morphine across four days, Animals in the NaBut treatment group received 250 mM NaBut b.i.d concurrently with the ramping dose of morphine b.i.d. Animals were assayed for thermal hyperalgesia by hot plate on day 5. Panel (**b**, **c**) male and female data from the thermal hyperalgesia hot plate assay comparing the effects of repeated administration of morphine vs repeated administration of morphine + 250 mM NaBut, and relevant controls (N = 14 per group Males and N = 5 Per Group Females). Animals treated with repeated administration of morphine showed a significant decrease in hot plate latency relative to the saline group (Males: morphine 14.8 s ± 0.6 vs saline 28.1 s ± 0.9, p < 0.001). (Females: morphine 18.9 s ± 2.8 vs saline 28.75 s ± 1.5 s, p < 0.001), indicating an increase in thermal hyperalgesia induced by repeated administration of morphine which was attenuated in the morphine + NaBut group. Panel (**d**) experimental timeline for reversal of hypersensitivity by NaBut. Animals received a ramping dose of morphine (i.p) as in panel (**a**), but only received NaBut treatment (p.o b.i.d) from day 5 onward. Panel (**e**, **f**) thermal hyperalgesia data from the three test days (Day 5, 7, 9) respectively. Animals receiving ramping dose morphine showed significant thermal hyperalgesia which was present on all three testing days, however animals which received ramping dose morphine + NaBut showed a significant decrease in their hyperalgesia by day 7, and was largely absent by day 9 (N = 9–10 per treatment group). Data were analyzed by two-way ANOVA with Bonferroni’s post-test (panel **b**) (F (1, 51) = 54.25 P < 0.001), panel (**c**) (F (1, 36) = 27.2 P < 0.001), panel (**e**) (F (1, 35) = 0.0540 P = 0.82, panel (**f**) (F (1, 35) = 9.32 P = 0.004), panel (**g**) (F (1, 35) = 4.02 P = 0.05)).

We and others have previously reported that chronic morphine exposure produces nociceptive hypersensitivity^5,34–36^. Consistent with those findings we found that a ramping dose of morphine produced a significant increase in nociceptive hypersensitivity as assayed by hot-plate latency relative to saline controls in both male and female mice. Male C57BL/6J mice receiving morphine for 4 days as described in the “Methods” and in Fig. 1a, had significantly reduced (P < 0.001) hot plate latency (14.8 s ± 0.6) vs saline control animals (28.1 s ± 0.9) (Fig. 1b). However, this increase in thermal hyperalgesia was prevented when male animals were concurrently treated with oral NaBut (b.i.d) which dose dependently reduced thermal hyperalgesia across all tested doses vs morphine alone (supplemental Fig. 1). Specifically, mice receiving 250 mM NaBut showed a significantly improved hot plate latency vs morphine alone (24.9 s ± 0.7) (P = 0.001) (Fig. 1b). We observed a similar reduction in hot plate latency for female mice induced by repeated administration of morphine relative to saline treated controls. Thermal hypersensitivity was significantly reduced in female mice treated with 250 mM NaBut and morphine concurrently (Fig. 1c). There were no significant sex differences between groups, and thus all subsequent experiments were conducted in male mice. To further characterize the effects of NaBut on the withdrawal induced hyperalgesia, we examined the potential efficacy of NaBut administration after the hyperalgesia had fully manifested. As with the previous experiments animals received repeated administration of a ramping dose of morphine, but received NaBut after manifestation of hypersensitivity on Day 5. (Fig. 1D). Male mice who received the ramping dose of morphine had developed significant (P < 0.001) thermal hyperalgesia by injection day 5 relative to saline (17.3 s ± 1.4 s Mor vs 26.1 s ± 0.9 s Sal), which was maintained throughout both test day 7 (18.99 ± 1.5 s Mor vs 27.7 ± 0.5 s Sal, P < 0.001) and test day 9 (21.5 s ± 2.0 s Mor, vs 27.63 ± 1.0 s Sal, P = 0.03). Animals in the NaBut treatment group developed significant hyperalgesia by day 5 (18.4 s ± 1.4 s Mor + But vs 26.7 s ± 0.7 s Sal + But, P < 0.001), however this was attenuated by test day 7 (24.4 s ± 1.5 s Mor + But, vs 25.9 s ± 1.1 s, P > 0.99).Opioid-induced hypersensitivity by repeated administration of morphine was prevented by co-administration of naloxone (Supplemental Fig. 2).

**Figure 2:**
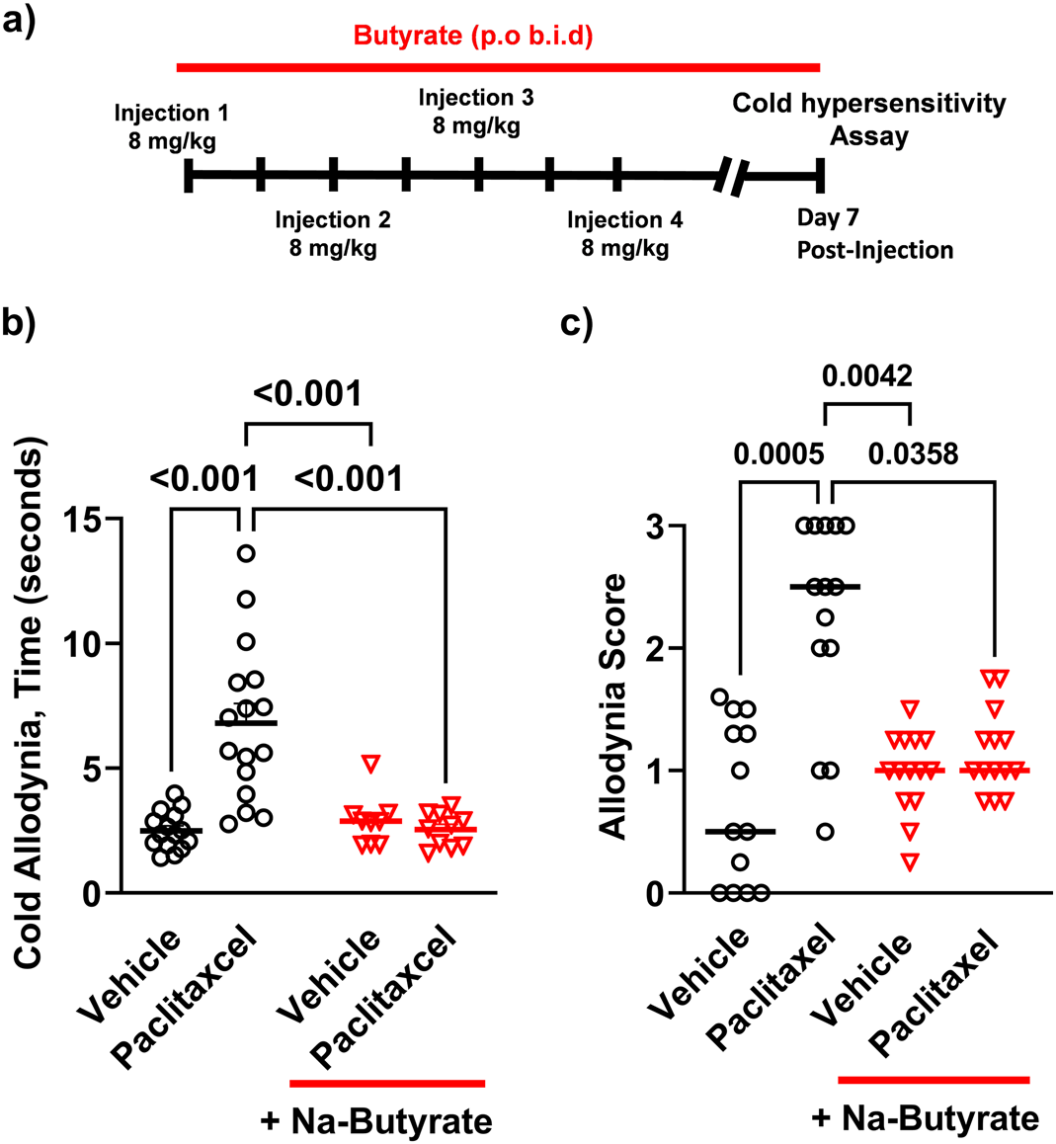
Paclitaxel-induced cold allodynia is alleviated by NaBut. (**a**) 8 mg/kg, i.p. of paclitaxel or vehicle (i.p.) injections on alternative successive days (1, 3, 5, 7) to a cumulative dose of 32 mg/kg (N = 11–15 per treatment group). Paclitaxel cohorts receiving the NaBut treatment were treated twice daily with a 250 mM oral gavage of NaBut for 14 days starting on the first day of paclitaxel i.p injection, (**b**) cumulative time spent engaging (flicking, shaking, grooming, licking etc.) the stimulated paw after acetone application (data analyzed by two-way ANOVA, F (1, 46) = 17.40 P < 0.001) (**c**) behavioral intensity scores from the acetone evaporation assay graded on a scale of 0–3 in increasing intensity (data analyzed by Kruskal–Wallis test with Dunn’s post-hoc P < 0.001). Animals treated with paclitaxel showed a significant increase in cold allodynia responses relative to the saline group, indicating an increase in peripheral behavioral hypersensitivity which was attenuated in the paclitaxel + NaBut group.

Various chemotherapeutics are known to cause profound peripheral hypersensitization in both human and rodents^37–39^. Paclitaxel has been shown to induce peripheral hypersensitization through a number of sensory modalities including thermal^8,27,40^. We investigated the effect of NaBut on paclitaxel-induced hypersensitivity. Male C57BL/6J mice received 8 mg/kg paclitaxel + vehicle every other day for 7 days, and then assessed for cold allodynia 7-days post final injection with an acetone evaporation assay (Fig. 2a). Paclitaxel treatment induced a significant increase in total time animals spent engaging the stimulated paw (6.9 s ± 0.6) (P < 0.001) when compared against animals that were treated with vehicle alone (2.5 ± 0.2). This enhanced cold hypersensitivity was significantly reduced by 250 mM NaBut treatment (2.5 s ± 0.2) (P < 0.001) (Fig. 2b). Animals were also scored for increasing intensity of aversive behaviors (See “Methods”) on a scale from 0 to 3 with 0 being the least aversive and 3 being the most. Paclitaxel treatment significantly increased aversive behavioral scores (2.2 ± 0.2) (P < 0.001) vs vehicle treatment alone (0.72 ± 0.18), and was not increased in the treatment group which received NaBut and paclitaxel concurrently (1.1 ± 0.3) (P = 0.036) (Fig. 2c) (data analyzed by Kruskal–Wallis test with Dunn’s post-hoc P < 0.001).

These data demonstrate that NaBut is able to prevent nociceptive hypersensitivity when given concurrently in both an opioid and chemotherapeutic model of nociceptive hypersensitivity. Furthermore, NaBut was an effective therapeutic for hyperalgesia following repeated administration of morphine.

### Repeated administration of morphine-induced neuronal hyperexcitability is attenuated by NaBut

**Figure 3:**
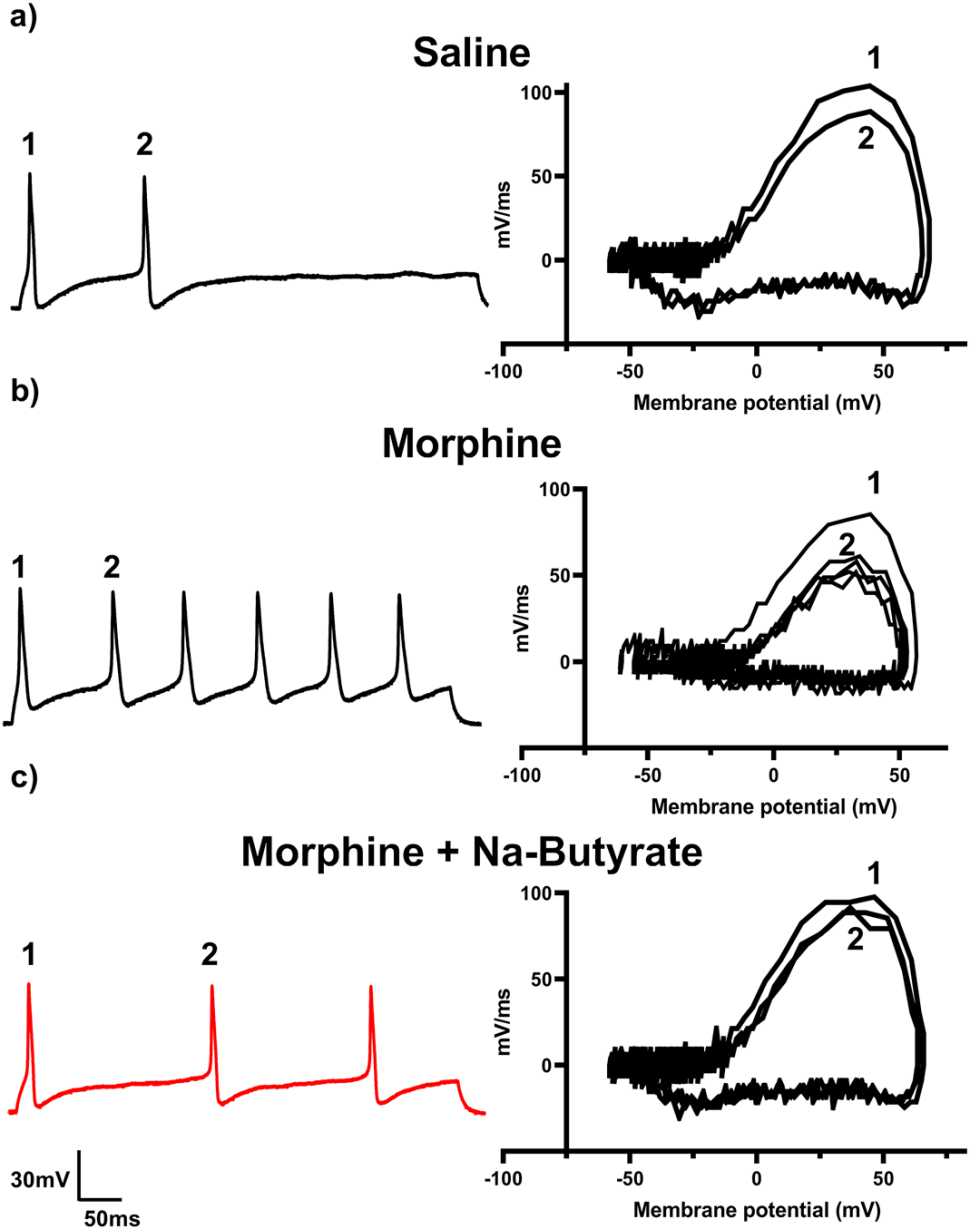
Repeated administration of morphine induces neuronal hyperexcitability and is attenuated by NaBut. (**a**–**c**) Representative neuronal action potential traces from isolated DRG neurons from Saline, morphine, and morphine + NaBut treated animals. The left panel shows the actional potential trace taken at 3× rheobase stimulation in a 500 ms pulse protocol, the right panel shows the associated phase plane analysis which is derived from the 1st derivative of the trace on the left, plotted against membrane potential. Repeated administration of morphine increased regenerative action potentials in the 500 ms pulse relative to saline indicating an enhanced neuronal excitability phenotype. This enhanced excitability is attenuated in the NaBut treatment group. Phase plane analysis of the action potential traces reveal a significant loss in maximum rate of membrane potential change during the rising phase of the action potential in the morphine group relative to saline controls, this change in maximum dV/dT is attenuated in the repeated dose morphine + NaBut group implicating voltage gated sodium channels in the potentiation of regenerative action potentials. Floating numbers (1, 2) indicate which action potential corresponds to which portion of the phase plane plots (which overlay one another), it should be noted that maximum rate of change (max dV/dT) occurs during the rising phase of the AP, and before the AP peak.

Chronic morphine treatment has been shown to enhance neuronal excitability and increase sodium currents^13,34,41,42^. We have previously demonstrated opioid induced changes to the gut microenvironment can be linked with secondary changes to neuronal excitability in the DRG^13^. Therefore, we investigated if neuronal changes would be similarly attenuated in those animals that received repeated administration of morphine (i.p) and NaBut (p.o, b.i.d). Animals received 4 days of a ramping dose of morphine outlined in Fig. 1a, and L4-S1 DRG collected on the 5th day for electrophysiological recordings in the current clamp configuration (See “Methods”). Representative results obtained from the 500 ms 10 pA step pulse recordings are shown in Fig. 3. Relative to saline controls, DRG neurons isolated from morphine treated mice produced significantly greater number of action potentials within the same recording period (Fig. 3a and b left). However, this enhanced neuronal excitability was attenuated in neurons isolated from NaBut treated mice (Fig. 3c left). Figure 3 also shows representative data from rate changes in membrane potential depolarization by phase plot analysis. Floating numbers on the pulse trace (Fig. 3b left) correspond to their associated phase plot trace, with the outermost trace being associated with the first action potential in the recording on the left panel (Fig. 3b right). We found the most relevant feature to be the distinct drop in the rate of dV/dT seen during the rising phase of the action potential (associated with the peak rate of change on the graph) in the repeated administration of morphine group (Fig. 3b right). Not only was this feature not seen in the saline control group, or the NaBut treatment group (Fig. 3a right), but it did not appear to greatly change between successive action potentials after the first evoked potential in a recording. This suggests that repeated administration of morphine induces a change in ion channel kinetics specific to the regenerating phase of the action potential, which is principally driven by voltage-gated sodium channels, and that NaBut prevents this change when given concurrently with morphine.

**Figure 4:**
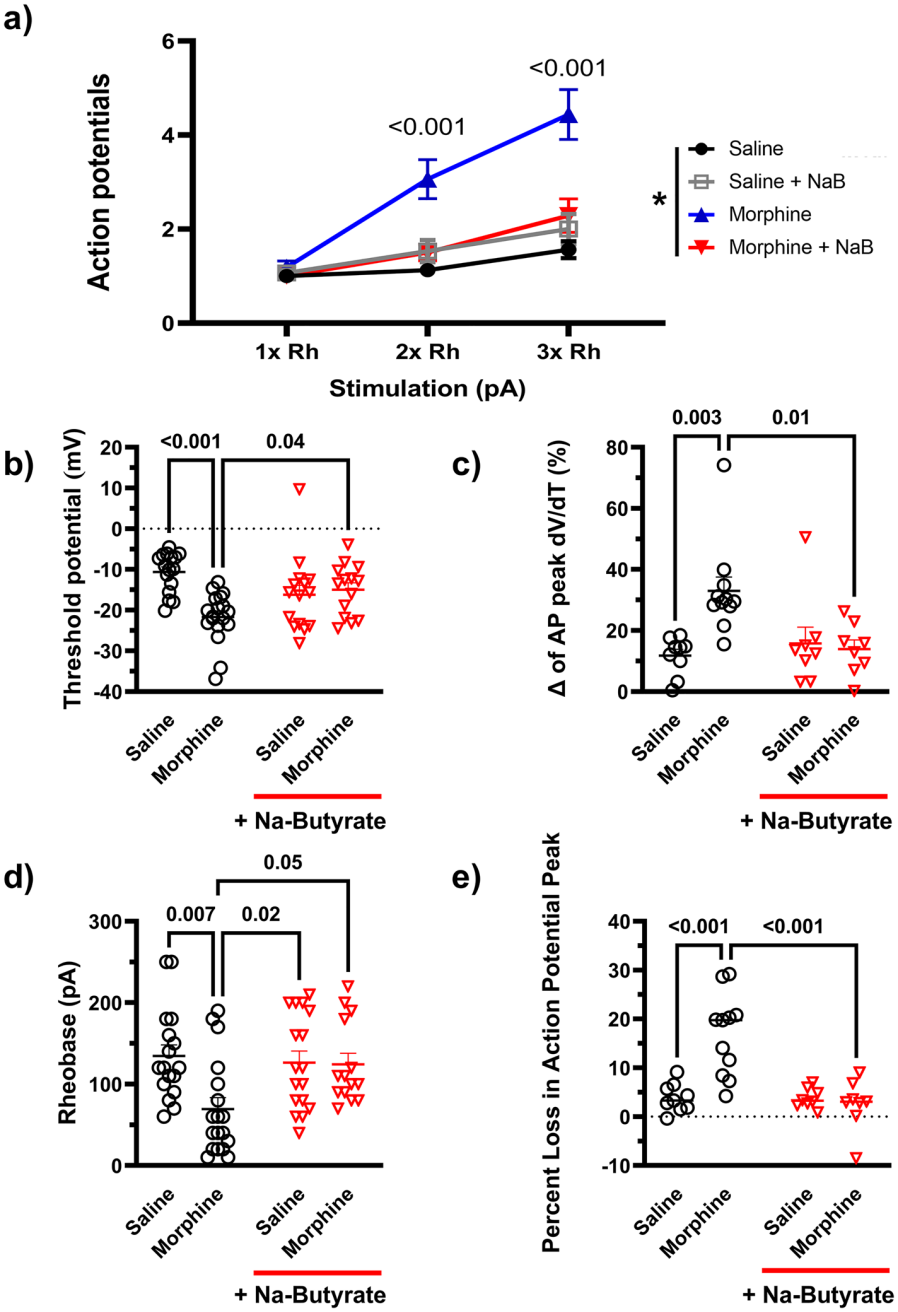
Repeated administration of morphine induces a hyperexcitable phenotype by altering threshold potential and rising phase kinetics of the neuronal action potential in DRG nociceptors. (**a**) Number of action potentials produced by isolated neurons at increasing levels of stimulation (1× Rheobase, 2× Rheobase, 3× Rheobase). Repeated morphine treatment significantly increases the number of action potentials seen in a 500 ms pulse period at 2× (3.0 ± 0.4 AP in 500 ms) and 3× (4.4 ± 0.5 AP) rheobase stimulation compared to saline controls (2× Rh P = 0.002, 3× Rh 2.3 ± 0.4 AP, P < 0.001), In-vivo treatment with NaBut attenuated these changes and reduced the number of action potentials significantly compared to morphine in the same pulse period (2× Rh 1.50 ± 0.2 AP P = 0.01, 3× Rh P < 0.001). (**b**) Threshold potential changes seen in isolated DRG neurons after repeated administration of morphine. Repeated administration of morphine induced a significant (P < 0.001) hyperpolarizing shift in the threshold potential relative to saline controls, In-vivo NaBut prevented this shift (P = 0.04). (**c**) Percentage loss of peak dV/dT in the phase plane analysis seen in Fig. 3. Repeated administration of morphine shows a significant (P = 0.003) loss in peak dV/dT between the first action potential and the subsequent regenerated AP’s in the trace. (**d**) Repeated administration of morphine lowers neuronal rheobase significantly (P = 0.007) but is recovered by the in-vivo treatment with NaBut (P = 0.05). (**e**) Percent loss in the AP peak measured in the phase plane analysis seen in Fig. 2. Repeated administration of morphine shows a significant (P < 0.001) loss in AP peak between the first action potential and the subsequent regenerated AP’s in the trace, this decrease in action potential peak is significantly attenuated in the NaBut treatment group (P < 0.001). Data were analyzed by Three-way ANOVA (F (1, 174) = 22.3 P < 0.001) in panel A, and by Two-way ANOVA with Bonferroni’s correction in panels (**b**–**e**). All data were collected using a 500 ms pulse protocol, N = 5–6, and n = 15–18 per treatment group.

We further quantified these changes in both the current pulse traces and phase plot analysis. Compared to the relevant controls, repeated administration of morphine produced a significant increase (P < 0.001) in the number of action potentials elicited in the same pulse period at both two times (2×) and three times (3×) the rheobase value (minimum current needed to illicit an action potential), and this enhanced neuronal excitability was significantly attenuated in the NaBut treatment group at both 2× (P = 0.004) and 3× rheobase respectively (P < 0.001) (Fig. 4a).

We also analyzed secondary characteristics of enhanced neuronal excitability including threshold potential, decrease in peak rate change from the phase plot analysis, rheobase and AP height. Threshold potential changes were observed in the repeated administration of morphine treatment group relative to saline controls. The repeated administration of morphine group had a significantly reduced (more negative) threshold potential (− 21.670 mV ± 1.44) compared to saline controls (− 10.60 mV ± 1.23) (P < 0.001), that was attenuated in the presence of NaBut treatment in-vivo (− 14.978 mV ± 1.693) (P = 0.04) (Fig. 4B). Quantification of the phase plot analysis revealed a significant drop in peak depolarization rate visualized in Fig. 3b. Compared to saline (11.734% loss ± 2.067), morphine treated animals had a significantly greater percent loss in peak membrane depolarization rate (mV/ms) between the first evoked potential and subsequent regenerative potentials (32.945% loss ± 4.537) (P = 0.003), but these kinetic changes were not seen in the NaBut treatment group (13.92% loss ± 2.98) (P = 0.01 vs Morphine) (Fig. 4c). Rheobase was similarly reduced in the repeated administration of morphine group (69.44 pA ± 13.90) (P = 0.007) relative to control (134.76 pA ± 13.48) and recovered in the NaBut treatment group (124.29 pA ± 12.95) (Fig. 4d). Lastly, we observed a significant decrease in action potential height between the first AP and the subsequent regenerative APs in the morphine treatment group. Compared to saline controls (3.9% decrease ± 0.9) the repeated administration of morphine group had a significantly (P < 0.001) greater percent decrease (16.742% decrease ± 2.51) in action potential height from the first evoked potential vs the second action potential (and subsequently equivalent regenerative potentials) in a pulse, however NaBut prevented this change in AP dynamics as well (2.50% decrease ± 1.82) (P < 0.001 vs morphine) (Fig. 4e).

These data suggest that repeated administration of morphine alters the electrical excitability of DRG neurons including changes in threshold, rheobase, and AP height resulting in enhanced firing frequency that is prevented by oral NaBut and likely related to underlying changes in those ion channels associated with the rising phase of the AP.

### Paclitaxel-induced neuronal hyperexcitability is attenuated by NaBut

**Figure 5:**
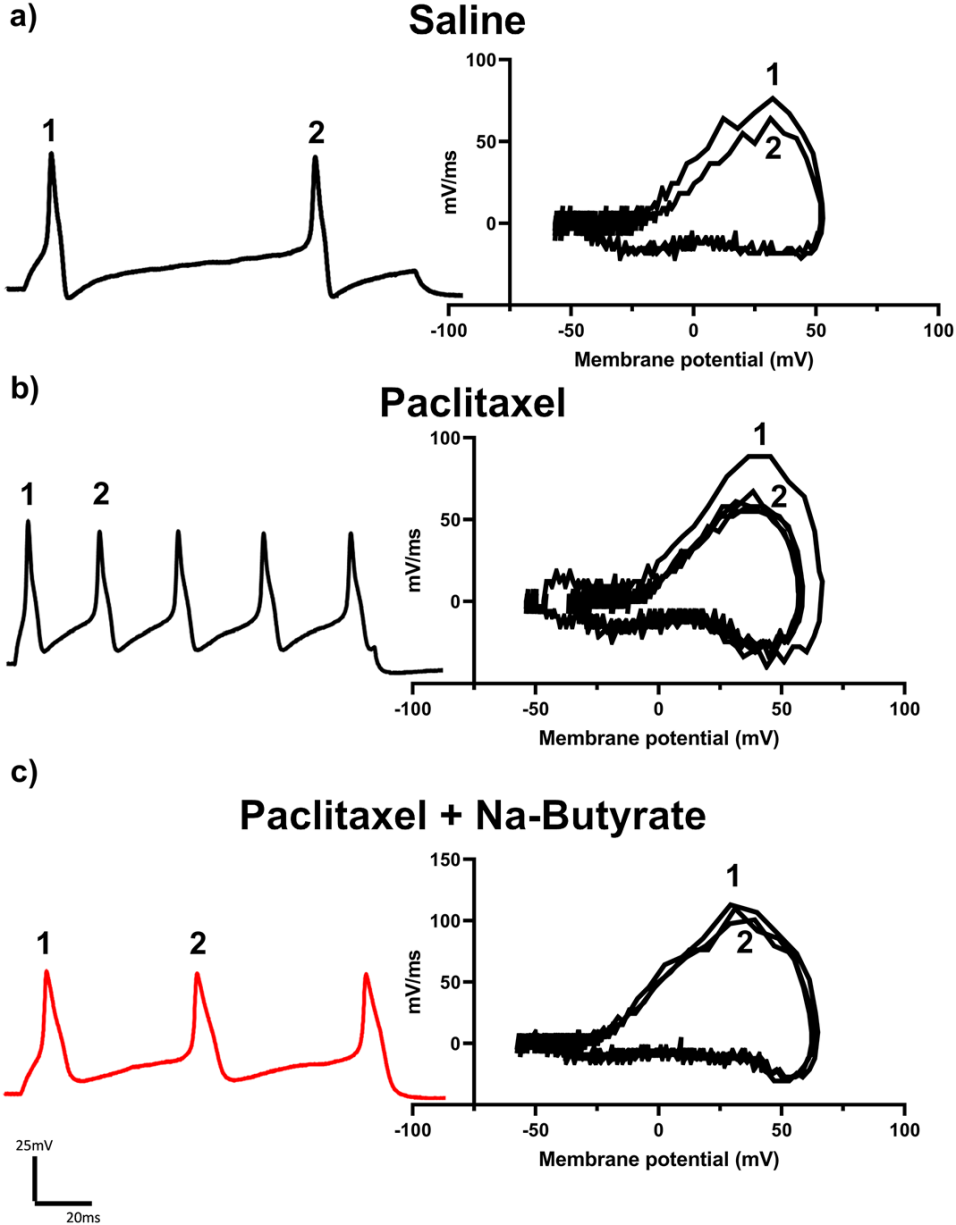
Paclitaxel-induced neuronal hyperexcitability is attenuated by NaBut. (**a**–**c**) Representative neuronal action potential traces from isolated DRG neurons from saline, paclitaxel, and paclitaxel + NaBut treated animals. The left shows the action potential trace taken at 3× rheobase stimulation in a 150 ms pulse protocol, the right shows the associated phase plane analysis which is derived from the 1st derivative of the trace on the left, plotted against membrane potential. paclitaxel treatment increased regenerative action potentials in the 150 ms pulse relative to saline indicating an enhanced neuronal excitability phenotype. This enhanced excitability is attenuated in the NaBut treatment group. Floating numbers (1, 2) indicate which action potential corresponds to which portion of the phase plane plots, which overlay one another.

**Figure 6:**
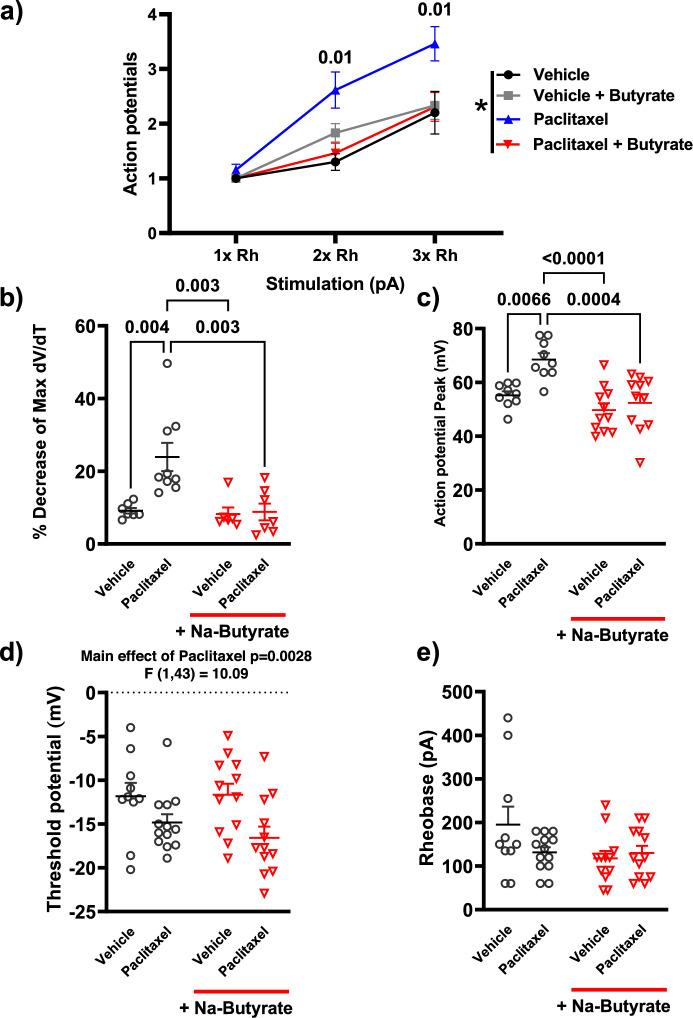
Paclitaxel induces a hyperexcitable phenotype by altering threshold potential and action potential peak in DRG neurons. (**a**) Number of action potentials produced by isolated neurons at increasing levels of stimulation (1× Rheobase, 2× Rheobase, 3× Rheobase). paclitaxel treatment significantly increases the number of action potentials seen in a 150 ms pulse period at 2× (2.65 AP’s ± 0.35) and 3× (3.46 AP’s ± 0.31) rheobase stimulation compared to saline controls (2× Rh P = 0.004, 3× Rh P = 0.008), In-vivo treatment with NaBut attenuated these changes and reduced the number of action potentials significantly compared to paclitaxel in the same pulse period (2× Rh 1.462 ± 0.18 P = 0.01, 3 × Rh 2.31 AP’s ± 0.26, P = 0.01). (**b**) Membrane potential shift between the evoked and regenerative potentials in a 150 ms pulse at 3× Rheobase. paclitaxel significantly altered the regenerative action potentials (P < 0.001), which was recovered in the presence of NaBut (P < 0.001). (**c**) Changes in Action potential peak height (mV) induced by paclitaxel (P+) and significantly reduced by the NaBut treatment condition (P+). (**d**) Threshold potential changes seen in isolated DRG neurons after paclitaxel treatment. Paclitaxel induced a significant (P = 0.0028) hyperpolarizing shift in the threshold potential relative to saline controls, In-vivo NaBut did not prevent this shift. (**e**) Action potential rheobase. Neither paclitaxel nor its vehicle significantly altered rheobase. Data were analyzed by Three-way ANOVA (F (1, 132) = 16.4 P < 0.001) in panel (**a**), and by Two-way ANOVA with Bonferroni’s correction in panels (**b**–**e**). Panel (**c**) (Two-way ANOVA, F (1, 36) = 4.31 P = 0.05), Panel (**d**): (Two-way ANOVA, F (1, 36) = 4.31 P = 0.05), Panel (**e**): (Two-way ANOVA, F (1, 43) = 3.137 P = 0.0836). All data were collected using a 150 ms pulse protocol, N = 3, and n = 10–13 per treatment group.

As expected from its established effects on peripheral behavioral sensitivity, paclitaxel is known to cause neuronal hyperexcitability^43^. Therefore, much like with morphine we wanted to investigate if NaBut (p.o, b.i.d) would be able to prevent paclitaxel (i.p) induced neuronal hyperexcitability in DRG neurons. Using the same treatment paradigm outlined in Fig. 2a, mice received 4 injections of 8 mg/kg paclitaxel and their DRG isolated 7 days post-final injection. Representative traces from the electrophysiological recordings of a 150 ms pulse at 3 × rheobase are shown in Fig. 5. Paclitaxel results bore a strong similarity to those obtained from repeated administration of morphine. Relative to the saline group, DRG of mice treated with paclitaxel in-vivo elicited more action potentials within the same pulse period and in-vivo treatment of NaBut was effective at attenuating this enhanced neuronal excitability (Fig. 5a–c, left). Phase plot analysis likewise indicated a significant drop in the mV/ms rate during the rising phase of subsequent regenerative action potentials (Fig. 5a–c, right). Additionally, enhanced firing frequency and changes in rising phase kinetics appear to be similar between both morphine and paclitaxel-induced neuronal excitability. Paclitaxel significantly (P = 0.01 vs vehicle) enhanced the number of action potentials recorded in a 150 ms pulse at 2× and 3× rheobase vs vehicle control, and NaBut attenuated this hyperexcitability at 2× (P = 0.01) and 3× rheobase (P = 0.01) (Fig. 6a). Consistent with the effects we observed in repeated administration of morphine (Fig. 4b), there was a similar reduction in depolarization rate (mV/ms) during the rising phase of the regenerative action potentials of neurons isolated from animals which were treated with paclitaxel (23.93% loss ± 3.88, P = 0.004 vs vehicle) (Fig. 6b). These changes in rising phase kinetics were not observed in either the vehicle controls (9.146% loss ± 0.74) or paclitaxel + NaBut treatment conditions (8.779% loss ± 2.32, P = 0.003) However, further quantification of the hyperexcitability induced by paclitaxel highlights several key differences between the two models. Unlike the morphine model where we only saw significant difference in AP height in successive and regenerative AP’s, paclitaxel induced a significant increase (68.51 mV ± 2.35, P = 0.006) vs vehicle (55.241 mV ± 1.48) in AP height independent of repetitive firing events. This enhanced excitability was none-the-less attenuated by NaBut treatment (52.37 mV ± 3.108, P < 0.001 vs paclitaxel) (Fig. 6c). Paclitaxel also induced a hyperpolarizing shift in the threshold potential which was not recovered by the NaBut treatment and was otherwise not significantly different from vehicle (Fig. 6d). Lastly, we did not see any significant difference in rheobases between any treatment condition with paclitaxel (Fig. 6e).

These data show that NaBut reduced hyperexcitability in neurons treated with paclitaxel. However, there were subtle differences between these changes and those induced by repeated administration of morphine.

### NaBut prevents Morphine-induced nociceptive hypersensitivity through a gut mediated mechanism

We next examined whether the effects of butyrate were mediated through a gut derived mechanism. For these studies, we investigated if conditioned media from colon tissues of morphine treated (as described above) mice enhance DRG neurons and if this was prevented by in-vivo treatment of NaBut (p.o, b.i.d).

A 0.5 cm section of the distal colon was dissected from mice treated with repeated administration of morphine and placed in growth media overnight, allowing the colonic milieu to diffuse. The enriched media was collected the next day and stored at − 80 °C. Following this, DRG neurons were isolated from naïve mice and incubated overnight with the colon condition media (CCM) media from morphine treated mice, after which neuronal excitability was measured.

**Figure 7:**
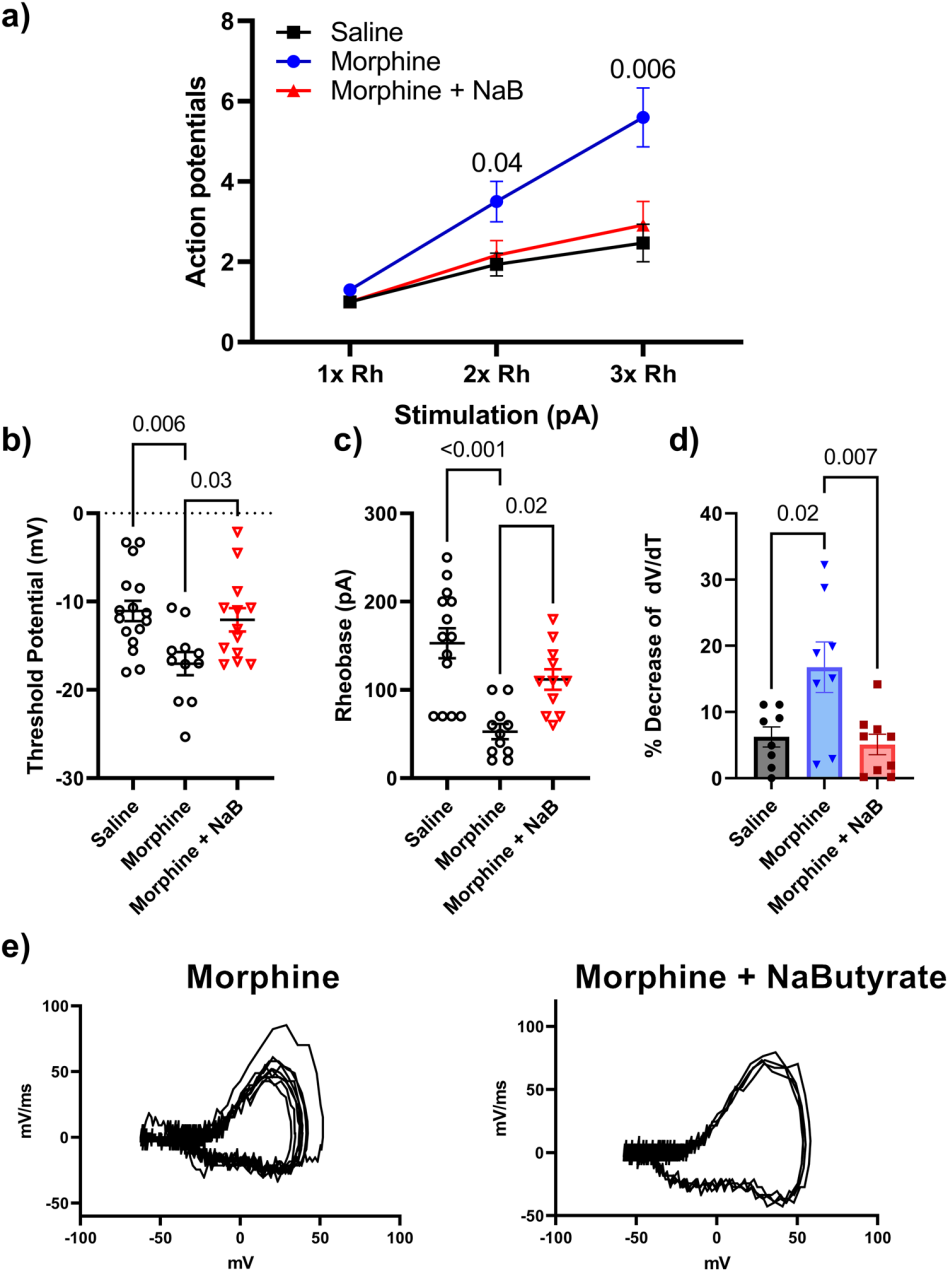
Repeated administration of morphine alters neuronal excitability through a gut mediated mechanism. (**a**) Number of action potentials produced at 1×, 2× and 3× rheobase (Ph) in isolated DRG neurons from a naïve animal. Neurons were treated with colonic conditioned media (CCM) which conveyed the corresponding phenotype seen in Fig. 3. The CCM from morphine treated mice significantly increases the number of action potentials seen in a 500 ms pulse period at 3× rheobase stimulation compared to saline (morphine 3.50 AP’s ± 0.5 vs saline 1.93 AP’s ± 0.2 at 2× rheobase P = 0.04 vs saline, and morphine 5.6 AP’ s ± 0.73 vs saline 2.47 AP’s ± 0.46 at 3 × rheobase P = 0.006 vs saline), this enhanced excitability was not observed in the naïve neurons treated with CCM from NaBut treated animals (2.91 AP’s ± 0.5 at 3× rheobase, P = 0.03 vs morphine). Panels (**b**–**d**) show the expected changes in threshold potential, and relevant kinetic changes observed in rheobase (52.7 pA ± 8.64. P < 0.001 vs saline) which was not significantly shifted by CCM isolated from morphine + NaBut treated animals (112 pA ± 11.6, P = 0.02 vs morphine). Indicating that the relevant phenotypes reported in Fig. 4 are likewise conveyed to the naïve neurons by morphine treated CCM but not in the presence of NaBut treated CCM. Panel (**e**) shows representative traces of Morphine, and Morphine + NaBut CCM treated naïve neurons, note the similarity in AP morphology to those representative traces in Fig. 2. All data were generated from naïve neurons recorded 24 h after adding CCM fractions to the cells. Data were data analyzed by Two-way ANOVA with Bonferroni’s post-test, F (4, 68) = 5.84 P < 0.001 (N = 4 n = 10–15 per group).

Figure 7 shows that naïve neurons that were incubated in CCM media isolated from morphine treated colon showed a significant increase in firing frequency at increasing levels of stimulation compared to those neurons (isolated from the same animal) that were exposed to saline CCM). The enhanced neuronal excitability was not seen with naïve cells incubated with morphine + NaBut CCM media (Fig. 7a). The threshold potential was shifted consistent with our expected results from the in-vivo experiments seen in Fig. 4. Morphine CCM treated naïve neurons showed a significant (P = 0.004) hyperpolarizing shift in the threshold potential (− 17.601 mV ± 1.3) vs saline CCM treated neurons (− 11.06 mV ± 1.1), this effect was attenuated in neurons treated with CCM isolated from morphine + NaBut treated animals (− 12.47 mV ± 1.29, P = 0.04 vs morphine) (Fig. 7B). Rheobase was similarly shifted towards a more excitable state by CCM media from morphine-treated mice, consistent with our findings from the in-vivo treatment experiments (Fig. 7c). Phase plot analysis of CCM treated cells revealed a similar pattern (compared to In-vivo data in Fig. 3) of rate (mV/ms) changes from the first evoked potential to subsequent regenerative AP’s during their rising phase from morphine CCM (16.77% decrease ± 3.81%, P = 0.02 vs saline), but not morphine + NaBut CCM treated naïve neurons (5.08% decrease ± 1.59%, P = 0.007) (Fig. 7d, e) (Data were analyzed by Two-Way ANOVA with Bonferroni’s post-test). We thus found that naïve neurons incubated with morphine treated CCM showed all the relevant electrophysiological outcomes we observed in the in-vivo treated experiments, which suggests that NaBut’s mechanism is at least in part emanating from its actions within the gastrointestinal tract.

**Figure 8:**
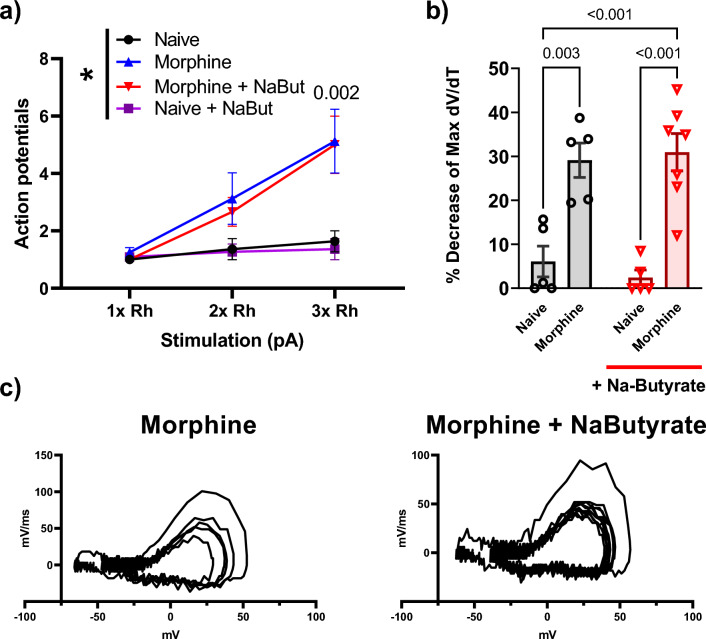
In-vitro morphine alters neuronal excitability and is not prevented by co-incubation with NaBut in isolated primary DRG neurons. (**a**) Number of action potentials produced by isolated neurons. Cells were exposed to 10 μM morphine, 10 μM morphine + 3 mM NaBut in-vitro, Naïve, or Naïve + 3 mM NaBut respectively. In-vitro morphine significantly increased number of action potentials fired in a 500 ms pulse (P = 0.001), which was not recovered by co-incubation with 3 mM NaBut (P = 0.001) when compared against the naïve control. (**b**) Percentage loss of peak dV/dT from phase plane analysis. (**c**) Representative phase plane analysis, note the differences from previous figures, NaBut had no significant impact on subsequent decreases in repetitive action potentials phase plane traces. data were analyzed by Two-way ANOVA F (1, 18) = 0.529 P = 0.48).

Finally, we investigated if NaBut would reduce neuronal excitability when applied directly to the DRG cell bodies. We isolated naïve DRG neurons from male C-57/Bl6 mice and incubated them overnight in culture media containing either 10 μM morphine or 10 μM morphine + 3 mM NaBut. Naïve neurons incubated with 10 μM morphine displayed significantly enhanced AP firing frequency in a 500 ms pulse (P = 0.002 at 3× Rheobase vs Saline) when compared to naïve control neurons from the same donor animal. This enhanced neuronal excitability was not attenuated by additional incubation with 3 mM NaBut (Fig. 8A). Furthermore, phase plot analysis revealed that 10 μM morphine induced the familiar decrease in maximum mV/ms rate during the rising phase of the regenerative AP’s relative to the naïve control, a feature that was also observed in the morphine + NaBut treatment group but not with NaBut alone (Fig. 8B). Representative phase plots are presented at the bottom of Fig. 8 (Fig. 8c). These data demonstrate that NaBut’s effects on neuronal hyperexcitability are associated with activity originating within the gastrointestinal tract rather than its potential effects directly at the level of the neuronal cell bodies.

## Discussion

The goal of our study was to investigate if oral supplementation with NaBut prevented drug induced nociceptive hypersensitivity. This was tested in two separate models, an opioid-induced model and a chemotherapy-induced hypersensitivity model. Through a combination of both whole animal behavioral assays and electrophysiological techniques, we determined that both repeated administration of morphine and paclitaxel treatment induced nociceptive hypersensitivity that can be prevented by treatment with NaBut. Additionally, we found that NaBut was effective at alleviating the withdrawal induced hyperalgesia after the behavioral nociceptive symptoms had manifested post-morphine treatment. These effects were also seen at the level of the dorsal root ganglia in the small diameter primary afferent nociceptors, where both in-vivo repeated administration of morphine, and paclitaxel-induced neuronal hyperexcitability was likewise attenuated by in-vivo NaBut co-treatment. Furthermore, both paclitaxel and repeated administration of morphine produced biophysical changes in the rising phase of regenerative action potentials. Additionally, we found that when neurons isolated from a naïve animal were incubated with media conditioned with the colonic milieu of an animal treated In-vivo with repeated administration of morphine, those naïve neurons take on the characteristic hyperexcitability. However, hyperexcitability was prevented when naïve neurons were incubated with the colonic milieu of animals treated with repeated administration of morphine and NaBut. Lastly, when we induced neuronal hyperexcitability in-vitro by way of incubating isolated naïve neurons with morphine, coincubation with NaBut did not prevent morphine-induced hyperexcitability, suggesting a gut-mediated rather than neuronally mediated for sodium butyrate’s underlying mechanism. It is noteworthy that while there are differences in the hyperalgesia associated with opioids and the chemotherapeutic agents, the hypersensitivity in both models is likely to involve an inflammatory response. The prevention by oral sodium butyrate suggest that development of an inflammatory response may underlie a gut mediated mechanism.

The anti-inflammatory properties of butyrate have been previously established by several studies over the last decade^9,44^. However, the mechanism(s) of action of how butyrate specifically reduces inflammatory consequences across a broad range of pathologies is not fully understood, although several key molecular targets have been identified^45,46^. Butyrate is a short chain fatty acid (SCFA) byproduct of bacterial fermentation of dietary fiber, and has been shown to engage several molecular targets and pathways that are known to reduce inflammation including gene expression, immune responses, oxidative stress pathways, and gut epithelial barrier function. Sodium butyrate’s ability to act as a histone deacetylase (HDAC) inhibitor has been well established. Consequently, butyrate’s role in altering gene expression and reducing pro-inflammatory cytokine production is an active area of research. Furthermore, butyrate has also been shown to modulate immune responses, specifically the activation and differentiation of immune cells, including T cells and macrophages both through HDAC and other molecular mechanisms^47^. NaBut can also act as an antioxidant, reducing oxidative stress, which is a key driver of inflammation, particularly regarding paclitaxel which primarily achieves its cytotoxicity by microtubule toxicity thereby enhancing oxidative damage^48^. Several studies have implicated the importance of butyrate as an essential component of gut barrier function which prevents the passage of harmful substances and bacteria into the systemic circulation, thereby reducing systemic and local inflammation^47,48^. Specifically, butyrate has been shown to increase the expression of tight junction proteins and stimulate the production of mucins^44,49^. Therefore, while the exact mechanism of how butyrate achieves its anti-inflammatory properties remains unclear, the existing evidence strongly suggests that these effects are primarily associated with the SCFAs interactions within the epithelium of the gut.

Butyrate levels are substantially reduced in fecal samples of opioid users^50^, Butyrate supplementation may also induce changes in the microbiome^33,51^. Further studies are needed to determine whether butyrate-induced changes in the microbiome directly impacts nociceptive hypersensitivity.

Our electrophysiological characterization of both morphine and paclitaxel demonstrated the expected increase in basal excitability in both animal models, consistent with the established literature, and this enhanced neuronal excitability was reduced by NaBut treatment. However, the specific mechanisms underlying both the enhanced excitability and the subsequent extinction of this effect by SCFA treatment may significantly differ between both morphine and paclitaxel. For example, previous studies by Li et. al.^52^ have demonstrated a strong association with voltage gated calcium channel (CaV3.2) changes induced by paclitaxel treatment. However, both drug models showed a similar increase in firing frequency and subsequent changes in regenerative action potential rising phase rates, both of which were attenuated by NaBut. This strongly suggests that both paclitaxel and morphine may (by potentially differential mechanisms) alter underlying sodium channel kinetics particularly those involved in the rising phase kinetics. In DRG neurons the predominating voltage gated sodium channel is NaV1.8 a TTX-R sodium channel, however NaV1.9 is also expressed ubiquitously in small diameter nociceptors and has been known to be involved in regenerative non-inactivating currents^53,54^. Future directions should include investigating specific changes in sodium channel conductance and kinetic changes, associated with both paclitaxel and repeated administration of morphine, including investigating specific sodium channel subtypes that may be driving the regenerative currents as well as the mechanism by which butyrate is attenuating these changes.

Our data also demonstrated that NaBut appears to achieve this reduced neuronal excitability through a gut mediated mechanism. The CCM experiments showed that the treatment condition the animal was exposed to (i.e. repeated administration of morphine, repeated administration of morphine + NaBut) defined the electrophysiological phenotype of the naïve neurons cultured in the colonic conditioned media. Conversely, when we incubated morphine and NaBut in-vitro, we found that morphine still produced enhanced neuronal excitability that was not responsive to treatment with NaBut. Taken together these data suggest that the SCFA does not directly affect somatic properties to such a degree as to attenuate the enhanced neuronal excitability. It is worth mentioning that we used a NaBut concentration which was previously established to induce HDAC inhibition (3 mM) for the in-vitro experiments^46,55^. There are several limitations to these interpretations, principally that the exact contents of the colonic milieu enriching the media were not characterized in this study. Furthermore, we also acknowledge that CCM experimental models were generated by injecting animals in-vivo, while the direct application of NaBut to morphine treated neurons was generated by an in-vitro approach, which raises the question of whether or not the excitability generated in-vivo is mechanistically the same or potentially induced by different mechanism, and therefore may not be responsive to SCFA treatment as a result. We feel that while that is valid, and the data should be taken with the proper context, chronic morphine’s effects on neuronal excitability have been well characterized utilizing both in-vivo and in-vitro approaches and these studies have found similar excitability changes to those reported previously^13,34^, and so we feel confident that while the underlying mechanisms may differ between in-vivo and in-vitro morphine the fact that such a high dose of NaBut had no effect on the underlying excitability strongly suggests a lack of direct efficacy on the neuronal physiology. However, we also acknowledge that our ex-vivo isolation technique removes any contributions from secondary cells such as glia that may contribute to the inflammatory microenvironment within the DRG, and previous literature particularly with paclitaxel has strongly implicated a glial component in the mechanism of CIPN^39,56^. Furthermore, a more thorough characterization of the morphological and histological changes that underlie both pre and post treatment in both drug models would greatly expand our understanding of the underlying mechanisms of NaBut’s therapeutic potential.

In conclusion, these data demonstrate that oral NaBut supplementation can prevent the nociceptive hypersensitivity induced by both repeated administration of morphine and paclitaxel. Additionally, our data suggests that preserving barrier function, butyrate may systematically affect neuronal function, and prevent damage of neuronal terminals exposed to the mucosal environment. Future studies into potential sex differences, different treatment models and more robust behavioral assays (i.e. prophylaxis vs treatment vs cotreatment) will greatly strengthen our understanding of the therapeutic value of SCFA treatment as an emerging and potentially valuable clinical tool for extending the already profound benefits of both opioids and chemotherapeutic drugs.

## Methods

### Animals

Adult male and female 8-weeks old C57BL/6J mice (Jackson Lab, Bar Harbor, ME) were used in this study. Mice were acclimatized at least 7 days before use and maintained throughout in standard housing conditions with access to food and water (24 ± 2 °C temperature; 50 ± 10% relative humidity). Animals were excluded from the study if they lost more than 20% of body weight, or developed an adverse reaction to injections over the course of treatment period. Investigators were blinded to the animal’s treatment condition during data collection for behavioral experiments.

### Repeated administration opioid model

Mice receiving morphine were injected with a ramping dose for 4 days starting at day one with 20 mg/kg intraperitoneally twice daily (i.p b.i.d), followed by 2 days of 40 mg/g i.p b.i.d, and a final day of 80 mg/kg i.p b.i.d morphine. Sodium butyrate (NaBut) (25–800 mM wt/v) was given by oral gavage twice daily (p.o b.i.d) for 4 days starting on the first day of morphine i.p injection. Mice in the therapeutic study, received morphine as described, but received the 250 mM sodium butyrate (p.o b.i.d) for four days starting on day 5. Mice in the naloxone cohort were injected with a ramping dose for four days starting at day one with 2 mg/kg i.p b.i.d, followed by two days of 4 mg/g i.p b.i.d, and a final day of 8 mg/kg i.p b.i.d naloxone.

### Chemotherapeutic model

For the studies on paclitaxel-induced hypersensitivity, adult male mice received 8 mg/kg, intraperitoneally (i.p) of paclitaxel or 1:1:18 vehicle (1 volume cremophor EL, 1 volume ethanol, 18 volume saline) i.p. injections on alternating days (days 1, 3, 5, 7) to a cumulative dose of 32 mg/kg as described previously^57^. Paclitaxel cohorts receiving the NaBut treatment were treated twice daily with a 250 mM oral gavage twice daily (p.o b.i.d) of NaBut for 14 days starting on the first day of paclitaxel i.p injection.

### Ethics declarations

All procedures and methodologies were conducted in accordance with the procedures reviewed and approved by the Institutional Animal Care and Use committee at Virginia Commonwealth University (VCU IACUC). All methods are reported in accordance with ARRIVE guidelines^58^.

#### Hot plate thermal hyperalgesia assay

Animals receiving morphine were tested on day 5,7, or 9. For Day 5, animals were tested 18 h after the last injection of morphine for the development of thermal hyperalgesia by utilizing a hot plate assay. The hot plate was set to 50 °$\mathrm{C}$ and animals were placed in the center of the heated area and allowed to engage in the assay for 30 s. During the assay if the animals engaged in hind paw licking, hind paw shaking, or jumping behaviors the assay was ended and the total time spent on the hot plate was recorded.

#### Acetone evaporation assay

Animals received 8 mg/kg paclitaxel or vehicle (1:1:18, cremophor, ethanol, saline) every other day for 7 days, and were subsequently tested for the development of cold allodynia by acetone evaporation. Acetone evaporation produces a non-noxious sensation of coolness approximately equivalent to 15–21 °C when applied to the skin^59^. Animals were tested at Day 7 and Day 14 post-paclitaxel treatment using a clear Plexiglas box with wire bottom to allow for access to the plantar surface of the animal’s hind paw. Animals were acclimated for approximately 1 h prior to testing after which time acetone was applied via a gavage needle to produce a drop (approximately 100 μL) onto the plantar surface of the left or right paw. Animal behavior was recorded for 30 s and the behavioral response graded on a scale of 0–3 in increasing intensity for both paws (i.e. 0, no response; 1, brisk withdrawal or flick of the paw; 2, licking of the paw; 3, prolonged licking/biting of the hind paw)^59^. Animals are then left undisturbed for 10 min and the trial repeated on the other paw. Scores were averaged across all trials to produce a single allodynia score. For comparison, 30 °C water was used as a control and applied to the paw and behaviors scored as above with acetone. the cumulative time spent engaging (flicking, shaking, grooming, licking etc.) the stimulated paw after acetone application was also quantified.

#### DRG isolation

Isolation of DRG neurons was carried out as described in previous studies^13,34^. Following sacrifice by cervical dislocation, DRG were harvested from spinal levels L5-S1 (those supplying the lower gastrointestinal tract) and immediately placed in cold (4 °C) Hanks’ Balanced Salt Solution (HBSS). Ganglia were then incubated (37 °C) for 18 min in HBSS with 36.6 μg/mL papain, washed with HBSS, and incubated for 1 h in HBSS with 1.5 mg/mL collagenase. Tissues were gently triturated and centrifuged for 5 min at 1000 rpm. The colonic media is decanted, and cells resuspended in neurobasal A medium containing 1% FBS, 1× B-27 supplement, 2 mM l-glutamine, 10 ng/mL GDNF, and penicillin/streptomycin/amphotericin B. The suspension was plated on poly-d-lysine/laminin coated coverslips and incubated (37 °C) for at least 24 h.

#### Electrophysiological recordings and solutions

Coverslips were transferred to a microscope stage plate and continuously perfused with external physiologic saline solution (ePSS). Standard ePSS contained the following in mM: 135 NaCl, 5.4 KCl, 0.33 NaH2PO4, 5 HEPES, 1 MgCl2, 2 CaCl2, and 5 glucose (pH adjusted to 7.4 with 1 M NaOH). DRG nociceptors of the small, C and Aδ fiber types^60^, low capacitance (< 30 pF) neurons were selected, and a GΩ seal achieved via pulled and fire-polished (2.5–4.5 MΩ) borosilicate glass capillaries. Standard internal PSS (iPSS) contains, in mM: 100 L-aspartic acid (K salt), 30 KCl, 4.5 Na2ATP, 1 MgCl2, 10 HEPES, 6 EGTA, and 0.5 NaGTP (pH adjusted to 7.2 with 3 M KOH). Recordings were made using a Axopatch 200B amplifier and Clampex/Clampfit 11.0 data acquisition software was used for analyses. Action potential (AP) threshold and rheobase estimates were measured from resting current, a 10pA stimulus pulse is applied in 10 pA steps starting from – 30 pA for 10 ms. For assessing multiple action potential firing events, a 500 ms (150 ms for paclitaxel) pulse period was used. Phase plots were generated by plotting the first derivative of the AP against membrane potential, maximum rate changes between successive potentials were measured during the rising phase of the AP.

#### Colon tissue conditioned media collection

In order to demonstrate whether inflammatory mediators released within the colon enhance neuronal excitability, we tested the effect of colon conditioned media (CCM) on DRG neuronal excitability, similar to previous studies^13^. Following sacrifice by cervical dislocation, full circumference colon segments 5 mm in length are resected and placed in 250 µL of neurobasal A medium containing 1% FBS, 1× B-27 supplement, 2 mM l-glutamine, 10 ng/mL GDNF, and penicillin/streptomycin/amphotericin B. The samples were incubated (37 °C) for 24 h and then the colon conditioned media was collected and either frozen in liquid nitrogen or transferred to freshly isolated naïve DRG neuron cultures. An additional 200 µL of fresh medium is added to the culture, which is then incubated (37 °C) for 24 h before performing electrophysiology experiments.

#### Data analysis

Sample sizes were determined using G*Power to detect 80% power for a medium effect size (Cohen’s d = 0.5) and an alpha level of 0.05. Data were analyzed using Graphpad Prism 9 (La Jolla, CA). Data were analyzed via a two-way ANOVA that included relevant drug treatment, and butyrate treatment as independent variables. Bonferroni’s post hoc test was used for those analyses which indicated a significant interaction (P < 0.05). A three-way ANOVA with Tukey’s post-hoc test was utilized when analyzing excitability data comparing increasing levels of stimulation against relevant drug treatment and butyrate treatment respectively on the number of evoked potentials.

### Supplementary Information

Supplementary Information 1.

Supplementary Figures.

## Data Availability

The datasets used and/or analyzed during the current study are available from the corresponding author on reasonable request. Source files are in supplementary files.
