# Supplementary material for: Short-chain fatty acid, butyrate prevents morphine-and paclitaxel-induced nociceptive hypersensitivity
Source: Sci Rep. 2023 Oct 18;13:17805. doi: 10.1038/s41598-023-44857-2 (PMC10584825; doi:10.1038/s41598-023-44857-2)
Supplement: Supplementary file 2 — Supplementary Figures. [file 41598_2023_44857_MOESM2_ESM.docx]

**Supplemental Figure 1. Sodium butyrate dose dependently reduces nociceptive hypersensitivity associated with the repeated administration of morphine.**

a) experimental timeline of injections. As detailed in figure 1 and methods, male animals were treated with a ramping dose model of morphine, and divided into different treatment groups and received 4 days of NaBut treatment at the given dose. Hot plate latency was 16.48s ± 0.64s morphine alone (N=26) (figure 1) (14.8s ± 0.6), 15.0s ± 2.9s morphine + 25mM NaBut (N=5), 21.0s ± 0.6s 80mM NaBut + morphine (N=5), 25.0s ± 1.2s 250mM NaBut + morphine (N=5), 30s ± 0.15s NaBut 800mM + morphine (N=5). These data indicate a dose dependent effect of NaBut on the nociceptive hypersensitivity induced by the repeated administration of morphine. Data were analyzed by one-way ANOVA with Bonferroni’s post-test,( F (3, 16) = 41.4 P<0.001) N=5 per group.

**Supplemental Figure 2. Naloxone coadministration blocks morphine-induced nociceptive hypersensitivity**

1. experimental timeline. All animals were treated with a ramping dose of morphine as detailed in the methods. Animals in the naloxone treatment group received a ramping dose concurrent with opioid treatment (2mg day 1, 4mg day 2-3, and 8 mg/kg day 4). b) hot plate latency data from the thermal hyperalgesia assay. Male animals receiving repeated administration of morphine showed a significant decrease in hot plate latency relative to the saline group (16.3s ± 0.7s Morphine vs 28.2s ± 0.8s Saline, P<0.001) indicating an increase in thermal hyperalgesia. Naloxone treatment effectively prevented morphine-induced thermal hyperalgesia from developing indicative of an opioid receptor dependent effect (16.3s ± 0.7s Morphine vs 26.270 ± 0.8s Morphine + Naloxone, P<0.001). Data were analyzed by two-way ANOVA with Bonferroni’s post-test, (F (1,16) = 27.4, P<0.001) N=5 per treatment group.
